# Outcomes of female fertility preservation with cryopreservation of oocytes or embryos in the Netherlands: a population-based study

**DOI:** 10.1093/humrep/deae243

**Published:** 2024-10-30

**Authors:** M Elena ter Welle-Butalid, Josien G Derhaag, Bo E van Bree, Ingeborg J H Vriens, Mariëtte Goddijn, Eva M E Balkenende, Catharina C M Beerendonk, Anna M E Bos, Irene Homminga, Sofie H Benneheij, H C van Os, Jesper M J Smeenk, Marieke O Verhoeven, Casandra C A W van Bavel, Vivianne C G Tjan-Heijnen, Ron J T van Golde

**Affiliations:** Department of Obstetrics and Gynecology, Maastricht University Medical Center, Maastricht, The Netherlands; GROW—School for Oncology and Reproduction, Maastricht University, Maastricht, The Netherlands; Department of Obstetrics and Gynecology, Maastricht University Medical Center, Maastricht, The Netherlands; GROW—School for Oncology and Reproduction, Maastricht University, Maastricht, The Netherlands; Department of Obstetrics and Gynecology, Maastricht University Medical Center, Maastricht, The Netherlands; GROW—School for Oncology and Reproduction, Maastricht University, Maastricht, The Netherlands; GROW—School for Oncology and Reproduction, Maastricht University, Maastricht, The Netherlands; Department of Internal Medicine, Division of Medical Oncology, Maastricht University Medical Center, Maastricht, The Netherlands; Center for Reproductive Medicine, Amsterdam UMC, Amsterdam, The Netherlands; Center for Reproductive Medicine, Amsterdam UMC, Amsterdam, The Netherlands; Department of Obstetrics and Gynaecology, Radboud University Medical Center, Nijmegen, The Netherlands; Department of Reproductive Medicine, University Medical Center Utrecht, Utrecht, The Netherlands; Center for Reproductive Medicine, University Medical Center Groningen, Groningen, The Netherlands; Division of Reproductive Endocrinology and Infertility, Department of Obstetrics and Gynaecology, Erasmus University Medical Centre, Rotterdam, The Netherlands; Department of Reproductive Medicine, Reinier de Graaf Hospital, Voorburg, The Netherlands; Department of Obstetrics and Gynaecology, Elisabeth-TweeSteden Hospital, Tilburg, The Netherlands; Center for Reproductive Medicine, Amsterdam UMC, Amsterdam, The Netherlands; Isala Fertility Centre, Isala Clinics, Zwolle, The Netherlands; GROW—School for Oncology and Reproduction, Maastricht University, Maastricht, The Netherlands; Department of Internal Medicine, Division of Medical Oncology, Maastricht University Medical Center, Maastricht, The Netherlands; Department of Obstetrics and Gynecology, Maastricht University Medical Center, Maastricht, The Netherlands; GROW—School for Oncology and Reproduction, Maastricht University, Maastricht, The Netherlands

**Keywords:** fertility preservation, oocyte cryopreservation, embryo cryopreservation, pregnancy, utilization rate

## Abstract

**STUDY QUESTION:**

What are the reproductive outcomes of patients who cryopreserved oocytes or embryos in the context of fertility preservation in the Netherlands?

**SUMMARY ANSWER:**

This study shows that after a 10-year follow-up period, the utilization rate to attempt pregnancy using cryopreserved oocytes or embryos was 25.5% and the cumulative live birth rate after embryo transfer was 34.6% per patient.

**WHAT IS KNOWN ALREADY:**

Fertility preservation by freezing oocytes or embryos is an established treatment for women with a risk of premature ovarian failure (caused by a benign or oncological disease) or physiological age-related fertility decline. Little is known about the success of cryopreservation, the utilization rate of oocytes or embryos, or the live birth rates.

**STUDY DESIGN, SIZE, DURATION:**

A retrospective observational study was performed in the Netherlands. Data were collected between 2017 and 2019 from 1112 women who cryopreserved oocytes or embryos more than 2 years ago in the context of fertility preservation in 10 IVF centers in the Netherlands.

**PARTICIPANTS/MATERIALS, SETTING, METHODS:**

A total of 1112 women were included in this study. Medical files and patient databases were used to extract data. Women were categorized based on indication of fertility preservation: oncological, benign, or non-medical. To indicate statistical differences the *t*-test or Mann–Whitney *U* test was used. Kaplan–Meier analyses were used for time endpoints, and log-rank analyses were used to assess statistical differences. The study protocol was approved by the medical ethics committee.

**MAIN RESULTS AND THE ROLE OF CHANCE:**

Fertility preservation cycles have been performed increasingly over the years in the Netherlands. In the first years, less than 10 cycles per year were performed, increasing to more than 300 cycles per year 10 years later. Initially, embryos were frozen in the context of fertility preservation. In later years, cryopreservation of oocytes became the standard approach. Cryopreservation of oocytes versus embryos resulted in comparable numbers of used embryos (median of 2) for transfer and comparable live birth rates (33.9% and 34.6%, respectively). The 5-year utilization rate was 12.3% and the 10-year utilization rate was 25.5%. The cumulative clinical pregnancy rate was 35.6% and the cumulative live birth rate was 34.6% per patient. Those who had fertility preservation due to benign diseases returned earlier to use their cryopreserved embryos or oocytes.

**LIMITATIONS, REASONS FOR CAUTION:**

The follow-up period after the fertility preservation procedure varied between patients in this study and not all frozen oocytes or embryos had been used at the end of this study. This might have led to underestimated outcomes reported in this study. Furthermore, intention to treat cannot be fully determined since women who started the fertility preservation procedure without success (cancellation due to low response) were not included in this study.

**WIDER IMPLICATIONS OF THE FINDINGS:**

This study provides data on the reproductive outcomes after various indications of fertility preservation. This knowledge can be informative for professionals and future patients to improve counseling and informed decision making regarding ovarian stimulation in the context of fertility preservation.

**STUDY FUNDING/COMPETING INTEREST(S):**

No funding was obtained for this study. The authors have no conflicts of interest to declare related to this study. V.T.H. received grants paid to the institute for studies outside the present work from AstraZeneca, Gilead, Novartis, Eli Lily, Pfizer, and Daiichi Sankyo. V.T.H. received consulting fees from Eli Lily outside the present work. M.G. received grants paid to the institute for studies outside the present work from Guerbet and Ferring. E.M.E.B. received a grant from The Dutch Network of Fertility Preservation for a study outside the present work.

**TRIAL REGISTRATION NUMBER:**

N/A.

## Introduction

Premature ovarian insufficiency is a clinical syndrome defined by the loss of ovarian activity before the age of 40. This can be caused by genetic predisposition, or by diseases diminishing the ovarian follicle pool either directly or indirectly through therapies used in oncological or benign diseases. Women who face a risk of premature ovarian insufficiency may consider a fertility preservation procedure to increase the likelihood of having their own genetic own children in the future. The American Society of Clinical Oncology, the International Society for Fertility Preservation, and the American Society for Reproductive Medicine recommend discussing fertility preservation with women who have cancer or benign conditions where treatment could possibly lead to premature ovarian insufficiency (Loren *et al.*, 2013; Martinez *et al.*, 2017; Oktay *et al.*, 2018).

Cryopreservation of oocytes and/or embryos after an IVF procedure is an established technique for preserving female fertility (Oktay *et al.*, 2018; Ter Welle-Butalid *et al.*, 2019). After retrieval, oocytes can be vitrified directly or fertilized to develop to an embryo before cryopreservation. The cryopreservation of oocytes instead of embryos provides patients autonomy on their later reproductive decisions, independent of the presence of a potential partner at the time of fertility preservation (Anderson *et al.*, 2020). Internationally, oocyte vitrification is considered a standard practice for fertility preservation for oncological reasons since 2012 (Loren *et al.*, 2013). In the Netherlands vitrification of oocytes was allowed and introduced in 2008. Prior to this period, oocytes could only be stored using slow-freezing techniques (Practice Committees of the American Society for Reproductive Medicine, Society for Assisted Reproductive Technology, 2013), but due to the low success rates with this technique, it was not employed as general practice in the Netherlands. Cryopreservation of oocytes and embryos for fertility preservation for medical reasons is covered by insurance in the Netherlands for women up to 42 years of age.

Various studies have looked at the utilization rates of banked oocytes/embryos and the fertility outcomes for cancer patients. A return rate for cryopreserved embryos was reported as between 9% and 63% (Oktay *et al.*, 2015; Hammarberg *et al.*, 2017; Ter Welle-Butalid *et al.*, 2019; Vriens *et al.*, 2020; Moravek *et al.*, 2021) and that for cryopreserved oocytes as between 0% and 5% (Balkenende *et al.*, 2018). Cumulative reported live birth rates after the use of cryopreserved embryos ranged between 9% and 75% and that for cryopreserved oocytes was between 33% and 50%. However, the number of patients in these studies was generally small, and the follow-up duration was short and different for studies on frozen embryos versus oocytes. Furthermore, in general, the follow-up period for frozen oocytes is shorter than that for embryos, which is probably due to the relatively recent introduction of this technique.

Additionally, women who face a risk of premature ovarian insufficiency and women who face physiological aging of their ovaries but want to postpone their child wish can consider fertility preservation. This latter situation is referred to as social freezing, or fertility preservation for a non-medical reason. From 2011, oocyte fertility preservation was offered in the Netherlands to women who are facing the physiological age-related fertility decline. This elective form of fertility preservation is not covered by insurance in the Netherlands. Women who opt for this form of fertility preservation are generally older and highly educated (Balkenende *et al.*, 2018). A recently published study showed a return rate of 27.4% after on average 39.9 months after non-medical cryopreservation (Loreti *et al.*, 2024).

Return rates for the various indications seem similar, however, the studies comparing them have had relatively short follow-up periods combined with low numbers of returning women.

Little is known about whether the results for cancer patients are different from those of women who have benign or non-medical indications for fertility preservation. The success rates for the oncological indications may be lower, but the existing literature to date is insufficient to determine this (Rodriguez-Wallberg *et al.*, 2019; Anderson *et al.*, 2020).

Therefore, we aimed to assess the use of cryopreserved oocytes and embryos and the associated pregnancy rates over a longer time period and with a minimum follow-up time of 2 years in the Netherlands. In this retrospective population-based cohort study, we categorized patients by indication for fertility preservation, that is, oncological, benign, or non-medical indication. Uptake and success rates were compared between the different indications. This evaluation of fertility preservation performance may be useful in counseling and optimizing decision making by patients considering IVF for fertility preservation reasons.

## Materials and methods

### Study population

This is a retrospective observational cohort study in the Netherlands. Ten out of 12 IVF centers in the Netherlands agreed to participate in the study. Data were collected from these IVF centers, which also participate in The Dutch Network of Fertility Preservation. The research population consists of women who had oocytes or embryos cryopreserved in the context of fertility preservation for cancer, benign diseases, or non-medical reasons. At time of data collection, we included patients who had stored oocytes or embryos for at least 2 years. Data were collected between 2017 and 2019. After the first analysis, missing data were collected up until 2023.

### Data source

In seven centers, data were manually collected from the medical files of individual patients. In three centers, a query was used to extract the data digitally from the patient files. The following data were collected and coded: general patient characteristics, indication of fertility preservation (oncological, benign, non-medical), number and date of fertility preservation cycles and stimulation protocol, number of oocytes or embryos retrieved and frozen, whether patients returned for embryo transfer (yes or no), date of return if applicable, number of embryo transfers, biochemical and clinical pregnancy rates, and live birth rate.

### Statistical analyses

Data analysis was performed using IBM SPSS Statistics, Armonk, NY, USA and StataCorp LLC, College Station, TX, USA. Patients were categorized per indication: oncological, benign, or non-medical. In addition, we performed explorative analyses on the ovarian stimulation protocols used: a GnRH antagonist protocol or a long GnRH agonist protocol. To indicate statistical differences for normally distributed continuous variables, *t*-test (*P* ≤ 0.05) was used. For non-normally distributed variables, the Mann–Whitney *U* test was used (*P* ≤ 0.05). For time endpoints, Kaplan–Meier analyses were performed and log-rank analyses were used for statistical analysis.

### Ethics committee approval

The study protocol was approved by the local medical ethics committee of Maastricht University Medical Center (project number 2017-0097). During the data collection, a new European law on privacy was implemented, the General Data Protection Regulation (GDPR). Therefore, in three centers, data were collected using a query to extract data from the database with pseudonymization of patient identification numbers.

## Results

### Patient demographics

**Figure 1.**
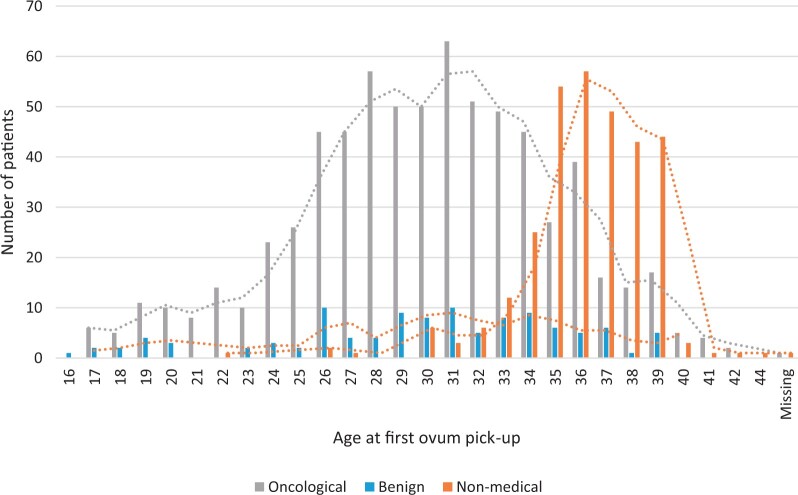
Distribution of age across the different indications.

**Figure 2.**
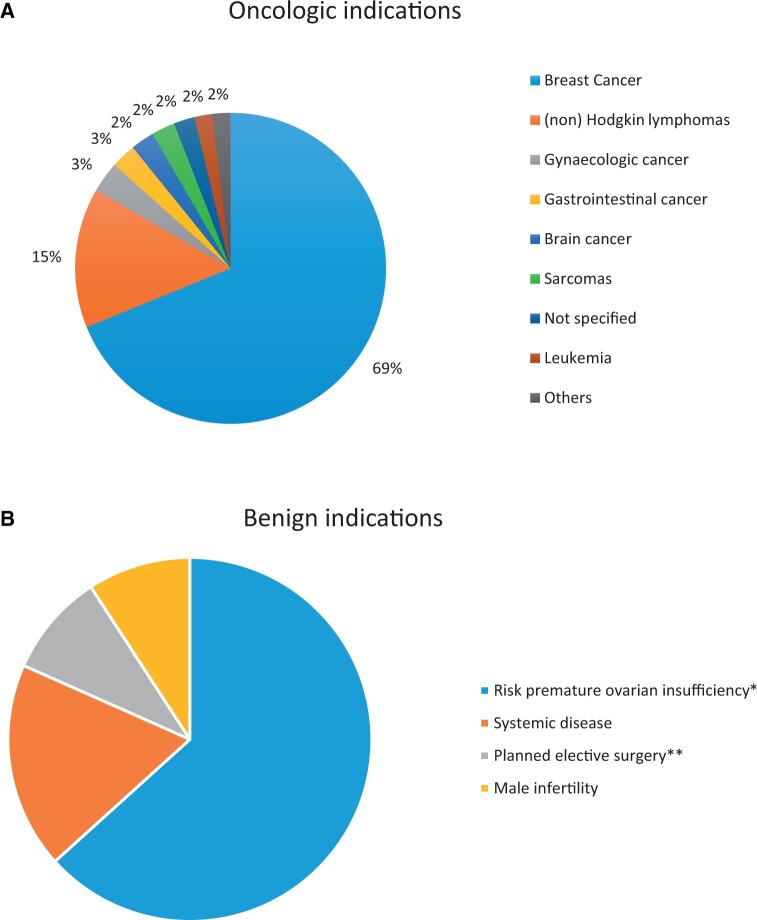
**Distribution of different indications. (A) Distribution of oncological indications; (B) distribution of benign indications.** *Due to genetic/familial predisposition, cysts/endometriosis and/or ovarian surgery, or Turner disease. **For *BRCA1* mutation carriers, transgender patients, and patients with uterus transplantation.

The study population consisted of data from 1112 women. This large population-based study included women who underwent a fertility preservation procedure between 2004 and 2019. Of these, 693 (62.3%) had undergone a fertility preservation procedure for an oncological reason, 109 (9.8%) had a benign reason, and 310 (27.9%) had a non-medical reason. Demographics are shown in Table 1 and Fig. 1, overall and per indication. In the oncological group, breast cancer was the most represented cancer type (68.8%, Fig. 2A). In the benign group, indications for fertility preservation varied from general risk of premature ovarian insufficiency (due to genetic predisposition), systemic disease (with possible gonadotoxic therapy), planned elective surgery (preventive bilateral salpingo-oophorectomy) or male infertility (Fig. 2B). The median follow-up period was 42 months (range 1–170).

**Table 1.** Demographic characteristics, *N* (%).

Indication for fertility preservation	Oncological 693 (62.3)	Benign 109 (9.8)	Non-medical 310 (27.9)	Total 1112
Female age at first oocyte retrieval—median and range	30 17–42	30 16–39	36 22–44	32
# children before FP				
One or more	97 (18.1)	7 (8.4)	7 (3.5)	111 (13.5)
None	438 (81.9)	76 (91.6)	195 (96.5)	709 (86.5)
Unknown	158 (22.8)	26 (23.9)	108 (34.8)	292 (26.3)
# pregnancies before FP				
One or more	149 (27.7)	15 (17.6)	30 (14.9)	194 (23.5)
None	389 (72.3)	70 (82.4)	172 (85.1)	631 (76.5)
Unknown	155 (22.4)	24 (22.0)	108 (34.8)	287 (25.8)

FP: fertility preservation.

The median age at first ovum retrieval was 32 years (range 16–44) in the whole group. Women in the oncological and benign groups were younger compared with those with a non-medical indication (median, 30 versus 36 years, *P* ≤ 0.05).

Of all patients with known information on pregnancy and children, 23.5% had been pregnant and 13.5% had one or more children before fertility preservation (Table 1). For the group with an oncological or benign indication, these rates were higher, and the ratio of live births to pregnancy rates seemed more favorable in comparison with the non-medical indication group.

### Cryopreservation of oocytes versus embryos

The median number of ovarian stimulation cycles was one per woman (range 1–7). In the benign and non-medical study groups, two or more stimulation cycles were performed more often (62.5% and 67.4%, respectively) compared with women with an oncological indication (20.6%) (*P* ≤ 0.05). There was no difference in number of oocytes retrieved using the GnRH antagonist protocol versus the long agonist protocol. The stimulation period of the GnRH antagonist protocol was 2 days shorter (11 versus 13 days, *P* ≤ 0.05) than the long agonist protocol (Supplementary Fig. S1).

Overall, for the majority of women oocytes were cryopreserved (*n* = 834, 75.0%) and for a minority of embryos (see Table 2). For a small group (*n* = 26), both oocytes and embryos were cryopreserved. Oocytes were cryopreserved for 61.8% of women with an oncological indication, for 88.1% of women with a benign indication, and for 100% of women with a non-medical indication.

**Table 2.** Oocyte retrieval yields and utilization rates, median (range).

Yield									Utilization rate	
	Patients (*N*)	Cycles per patient	Oocytes retrieved per patient	Frozen oocytes per patient	Thawed oocytes per patient	Oocytes eligible for ICSI after thawing	Fertilized oocytes	Frozen embryos	Embryos eligible for embryo transfer	Embryos used for embryo transfer
**Total**										
Oocytes	834	1 (1–7)	16 (1–66)	13 (1–52)	–	–	–	–	–	–
*Returned*	75	2 (1–5)	16 (2–51)	13 (1–42)	9 (1–30)	7 (1–27)	4 (0–19)	–	3 (0–13)	2 (0–9)
*Not returned*	759	1 (1–7)	16 (1–66)	13 (1–52)	–	–	–	–	–	–
Embryos	252	1 (1–3)	12 (1–52)	–	–	–	6 (1–43)	6 (1–32)	–	–
*Returned*	39	1 (1–3)	13 (3–42)	–	–	–	8 (1–29)	6 (1–20)	4 (0–18)	2 (0–9)
*Not returned*	213	1 (1–3)	11 (1–52)	–	–	–	6 (1–43)	6 (1–32)	–	–
**Per indication**										
Oocytes										
** Oncological**	428	1 (1–5)	14 (1–56)	11 (1–45)	–	–	–	–	–	–
*Returned*	26	1 (1–3)	13 (3–40)	13 (2–36)	9 (2–24)	7.5 (2–16)	4 (1–12)	–	3 (1–8)	2 (1–5)
*Not returned*	402	1 (1–5)	14 (1–56)	11 (1–45)	–	–	–	–	–	–
** Benign**	96	2 (1–5)	19 (1–57)	12 (1–45)	–	–	–	–	–	–
*Returned*	15	1 (1–3)	11 (2–43)	10 (1–33)	10 (1–20)	7 (1–17)	4 (1–12)	–	2 (1–9)	1 (1–5)
*Not returned*	81	1 (1–5)	20 (1–57)	15 (1–45)	–	–	–	–	–	–
** Non-medical**	310	2 (1–7)	21 (1–66)	17 (1–52)	–	–	–	–	–	–
*Returned*	34	2 (1–5)	22 (2–51)	18 (2–42)	9 (2–30)	7 (2–27)	4.5 (0–19)	–	3 (0–13)	2 (0–9)
*Not returned*	276	2 (1–7)	21 (1–66)	17 (1–52)	–	–	–	–	–	–
Embryos										
** Oncological**	242	1 (1–3)	11 (1–52)	–	–	–	6 (1–43)	6 (1–32)	–	–
*Returned*	36	1 (1–3)	12 (3–30)	–	–	–	7.5 (1–18)	6 (1–18)	4 (0–18)	2 (0–9)
*Not returned*	206	1 (1–3)	11 (1–52)	–	–	–	6 (1–43)	6 (1–32)	–	–
** Benign**	10	1 (1–3)	17 (8–42)	–	–	–	7 (2–29)	6.5 (3–20)	–	–
*Returned*	3	1 (1–1)	33 (21–42)	–	–	–	17 (15–29)	14 (13–20)	16 (14–18)^a^	3 (2–5)
*Not returned*	7	1 (1–3)	11 (8–26)	–	–	–	7 (2–15)	5 (3–12)	–	–
** Non-medical**	0	–	–	–	–	–	–	–	–	–
*Returned*	0	–	–	–	–	–	–	–	–	–
*Not returned*	0	–	–	–	–	–	–	–	–	–

a Number of eligible embryos for one patient not available.

In the entire study population, the median number of oocytes cryopreserved was 13 (range 1–52), and the mean number of embryos cryopreserved was 6 (range 1–32) per woman. This resulted in a comparable median number of embryos used for embryo transfer, which was 2 (range 0–9) for both groups. In both groups, more embryos were eligible for transfer than were used eventually. In five cases (4.4%), no embryos were suitable for transfer. The median number of oocytes cryopreserved in the oncological group was 11 (range 1–45) and in the benign group, it was 12 (range 1–45), which was slightly lower than in the non-medical group (median 17, 1–52) (*P* ≤ 0.05). Finally, the number of embryos eligible for embryo transfer did not differ per indication group (Table 2).

**Figure 3.**
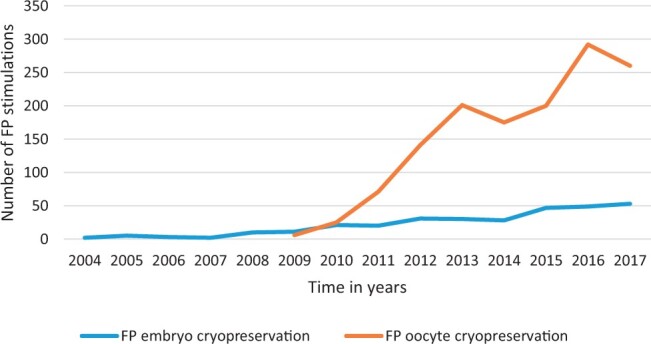
Number of fertility preservation cycles with cryopreservation of embryos and oocytes in the Netherlands.

In 2009, the first oocyte cryopreservation procedures were performed and, since then, the number of fertility preservation procedures rapidly increased in our study population (Fig. 3). Especially the number of procedures using oocyte cryopreservation strongly increased, resulting in more than 250 oocyte cryopreservation procedures and only about 50 embryo cryopreservation procedures in 2017.

### Utilization rates

Of the women who returned (*n* = 114), 33.1% had cryopreserved embryos, 63.6% had cryopreserved oocytes, and 3.4% (*n* = 4) had cryopreserved embryos and oocytes (Table 2).

**Figure 4.**
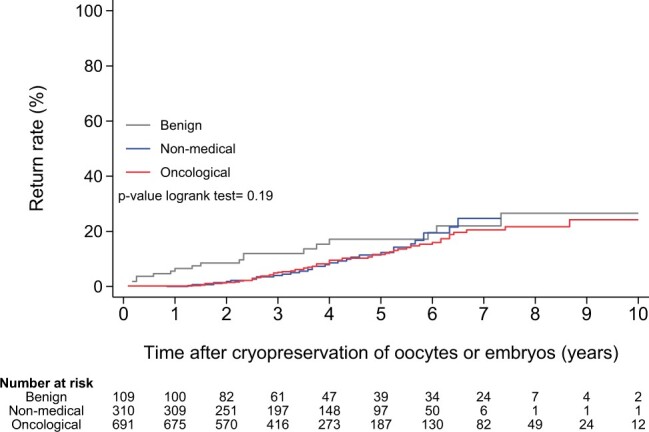
Cumulative utilization rate after fertility preservation.

The return rates over time per indication are shown in Fig. 4. The median period of those who returned after cryopreservation to attempt to become pregnant was 42 months (range 1–104). The 5-year return rate was 12.3% for the whole cohort, increasing to 25.5% after 10 years. In comparison with the oncological and the non-medical group, women with a benign indication used their cryopreserved oocytes or embryos relatively more often within 5 years (17.1%, *P* ≤ 0.05), with a median period of 23 months (range 2–88). However, from 6 years onwards, the return rates from the oncological and non-medical groups increased to percentages similar to the benign group.

### Success rates

**Figure 5.**
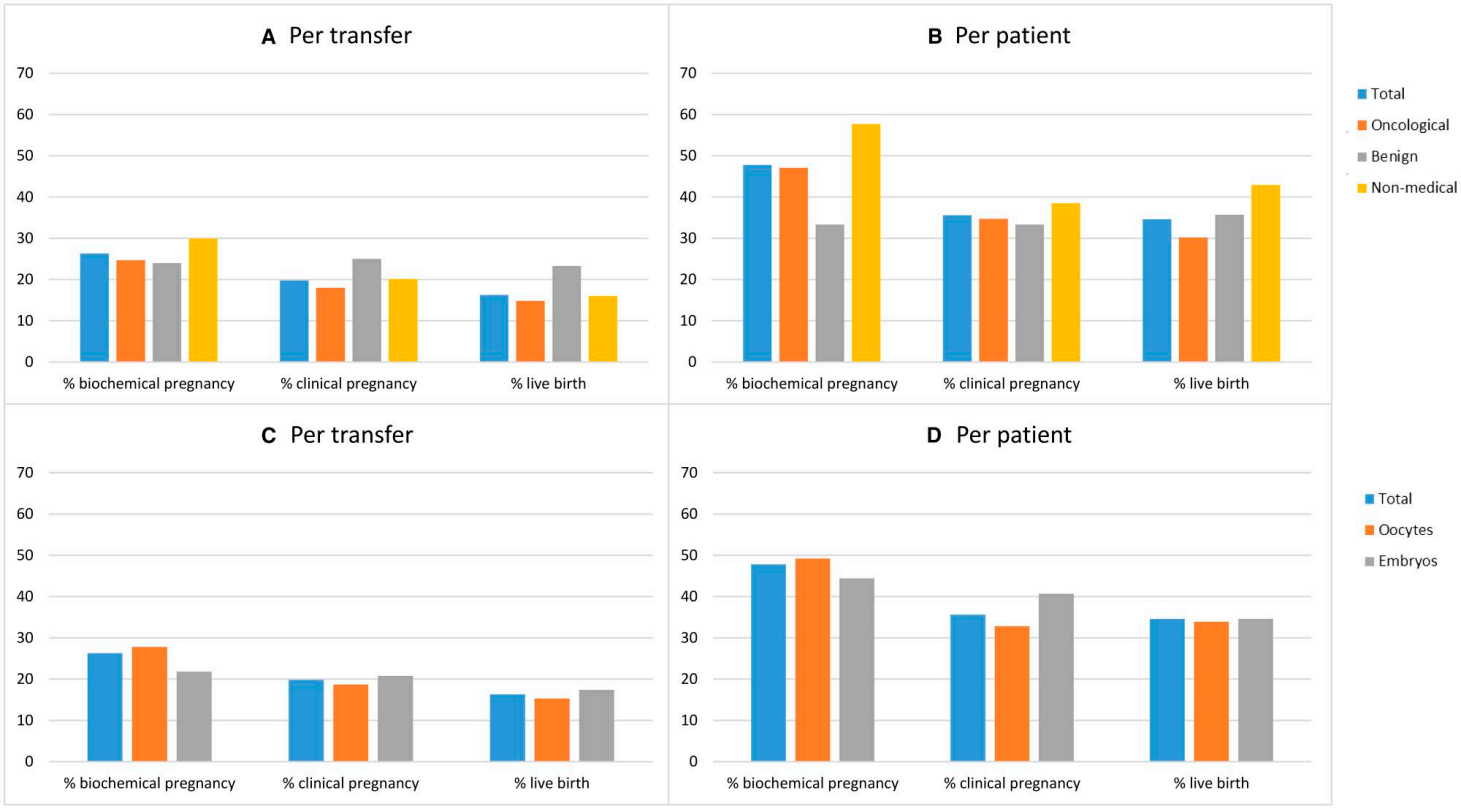
**Success rates per embryo transfer and per patient.** (**A**) Success rates per embryo transfer divided per patient subgroup. (**B**) Cumulative success rates per patient divided per patient subgroup. (**C**) Success rates per embryo transfer divided per method of fertility preservation. (**D**) Cumulative success rates per patient divided per method of fertility preservation.

In total, 205 embryo transfer procedures in 96 patients were performed (Fig. 5), with an average of 1.08 ± 0.3 embryos per transfer procedure. Success rates of all performed embryo transfers are shown as biochemical pregnancy rate, clinical pregnancy rate, and live birth rate (Fig. 5). These numbers are shown per subgroup (Fig. 5A and B) and per method of cryopreservation (Fig. 5C and D).

In the total study population, the biochemical pregnancy rate was 26.3%, the clinical pregnancy rate 19.8%, and the live birth rate 16.3% per embryo transfer (Fig. 5A). The benign group had the highest live birth rate per embryo transfer, that is, 23.3% for the benign group versus 14.8% for the oncological group and 16.0% for the non-medical group (*P* = 0.61).

Per patient, the biochemical pregnancy rate was 47.8%, the clinical pregnancy rate was 35.6% and the live birth rate was 34.6% (Fig. 5B). The live birth rate was lowest for the oncological indications (30.2%), followed by the benign group (35.7%), and highest for the non-medical group (42.9%), although differences were not statistically significant (*P* = 0.59). The live birth rate per patient was 33.9% for those who had cryopreservation of oocytes compared with 34.6% (*P* = 0.46) for the patients with cryopreserved embryos. Live birth rates were also evaluated in different age groups per indication. However, the numbers were too small to find statistical differences. In addition, age was divided differently for the different indications, so it was not possible to make a comparative analysis by age group.

## Discussion

Our study provides data on the reproductive outcomes of female fertility preservation procedures of 1112 women in the Netherlands. In the early years, mainly embryos were frozen, whereas during more recent years cryopreservation of oocytes became the preferred option. We show that the number of embryos used for transfer originating from cryopreserved oocytes was comparable with the numbers from those with cryopreserved embryos and that the live birth rates after using cryopreserved oocytes or embryos were the same. Therefore, we can conclude that the cryopreservation of oocytes seems equally effective as compared to freezing embryos. After 10 years of follow-up, about a quarter of women had returned to use their cryopreserved oocytes or embryos, which was not different between the indication groups. Overall, embryo transfer resulted in a live birth rate of 35% per patient with a median of two transferred embryos per patient. Noteworthy, in two-thirds of women who had returned, oocytes had been cryopreserved.

We included women over a long period of time. Over the years, technology and protocols have evolved. In 2004, cryopreservation of oocytes was less successful than nowadays (and therefore not applied in the Netherlands) (Practice Committees of the American Society for Reproductive Medicine, Society for Assisted Reproductive Technology, 2013). However, since 2008, a new freezing method, i.e. vitrification of oocytes was introduced and gradually became a new standard in the Netherlands. This study has shown that the majority of women who returned had undergone oocyte cryopreservation, even though their follow-up time was on average shorter than those who underwent embryo cryopreservation. By cryopreserving oocytes, the patient remains autonomous on her reproductive decisions in the future (Mertes and Pennings, 2012; DeVore *et al.*, 2019). In contrast to an IVF procedure for subfertility, the women who undergo fertility preservation for future use may have another or no partner at the time they wish to fulfill their family planning (Rodriguez-Wallberg *et al.*, 2019). If, in this situation, embryos are cryopreserved with a former partner, they cannot be used without permission of this former partner (Wyndham *et al.*, 2012). Hence, we believe that because of the same efficiency in terms of pregnancy for oocytes and embryos, cryopreservation of oocytes could be recommended.

The number of oocytes retrieved was not related to the ovarian stimulation protocol used (data not shown), in line with results reported by others (Bosch *et al.*, 2020). However, the median procedure was on average 2 days shorter for the GnRH antagonist protocol than for the long agonist protocol. Since it is important that the fertility preservation procedure for oncological indications is achieved with a minimal delay in the start of cancer treatment, short antagonist protocols are currently the preferred strategy (Anderson *et al.*, 2020). A shorter ovarian stimulation is also beneficial if two ovarian stimulation procedures can be considered for women who have enough time available before starting their oncological treatment.

Women in the non-medical group had a higher total number of oocytes cryopreserved compared to the women in the medical group. Of note, women in the non-medical group generally underwent more than one stimulation cycle for fertility preservation. In the medical group, this was rarely the case, because of time restraints due to the need to start gonadotoxic treatment. This time restraint could also occur in the benign group if systemic treatment is planned. Despite the lower number of oocytes, the medical group had a comparable number of embryos available for transfer as the non-medical group. It could be reasoned that due to the older age (especially from 38 years of age) in the non-medical group, more oocytes are needed for one suitable embryo for transfer (Stoop *et al.*, 2012; Cobo *et al.*, 2021).

After a follow-up period of 10 years, a quarter of women had returned to utilize the banked oocytes and embryos. The utilization rate was not dependent on the underlying reason for fertility preservation. This utilization rate is comparable with the percentages reported by others, as reviewed by us (Ter Welle-Butalid *et al.*, 2019). These earlier studies were generally published 1–4 years after cryopreservation and had a low number of fertility preservation cycles, which was considered a good reason for the low return rates. In this current study, the follow-up time was longer and the number of fertility preservation procedures was higher. Nevertheless, the utilization rate we saw after 10 years was also comparable with earlier reports. In the medical group, one of the reasons not to return might have been the recovery of ovarian function in the first years after the cytotoxic treatment with restoration of fertility. Actually, 91.3% of women treated with chemotherapy for breast cancer below the age of 40 do have a recovery of ovarian function (Vriens *et al.*, 2020; Abel *et al.*, 2021; Marklund *et al.*, 2021). It could be that spontaneous conception is more successful in this group. It could also be that these women have fertility treatments such as intrauterine insemination or fresh IVF cycles (Balkenende *et al.*, 2018; Loreti *et al.*, 2024). For others, the child’s wish may have turned out to be bygone (Martin-Babau *et al.*, 2018). Another reason could be that they were not able to return because of their current medical condition or that they are deceased. Others might postpone the fulfillment of their child’s wish due to adjuvant endocrine therapy (Partridge *et al.*, 2023; Ter Welle-Butalid *et al.*, 2023). Some may not have returned because of the cryopreservation of embryos with a former partner. If true, the return rate may yet increase with longer follow-up for the women who had undergone oocyte preservation.

There was a trend in the data showing that the benign group tried to achieve pregnancy earlier with cryopreserved oocytes or embryos than patients with an oncological indication (median period 23 versus 42 months, respectively). This might originate from the fact that patients in the oncological group are recommended to postpone a pregnancy for a certain period (Peccatori *et al.*, 2013). Therefore, it is not expected that women return within this timeframe after cancer treatment (Vriens *et al.*, 2020).

The present study shows that fertility preservation through freezing oocytes or embryos in women with cancer or other risks for fertility loss is a strategy which can result in pregnancies and in healthy genetic own babies being born. The overall live birth rates per woman found in this study are comparable to previous reports (Cobo *et al*., 2018; Tsafrir *et al.* 2022; Loreti *et al.*, 2024). Rodriguez-Wallberg *et al.* (2019) showed higher live birth rates for the benign indications (47%), which could be explained by the younger population of their study (mean 27.1 years). Their live birth rate per patient for the oncological indications was lower (21%), which could be explained by a different distribution of malignant diseases in their population. The live birth rates per transfer in our study were lower compared to the more recent results of Loreti *et al.* (2024). This could be due to the lower amount of double embryo transfers in our study population, which could be explained by the Dutch regulations that only allow double embryo transfers from the age of 38 years. Women with a medical indication were younger and had more often former pregnancies than those with a non-medical indication. These differences seem logical because the reason for the initiation of the fertility preservation procedure is different between both groups. The high representation of breast cancer patients in the oncological group can be explained by the fact that breast cancer is the most common malignancy in women of reproductive age (Lawrenz *et al.*, 2011; Rodriguez-Wallberg *et al.*, 2019). The live birth rate per embryo transfer and per patient seemed to be lower for the oncological group. Due to the low numbers, live births could not be comparatively analyzed per age group across the indications. It is noteworthy, that the significantly younger oncological group, has lower poorer than the older non-medical group. In agreement with our results, Rodriguez-Wallberg *et al.* (2019) also showed a lower live birth rate per transfer in the oncological group. It could be reasoned that despite the use of cryopreserved embryos that are not exposed to gonadotoxic therapy, the fertility of the women is still impaired due to prior oncological therapy. Receptivity of the endometrium or hormonal regulation patterns might still be disturbed. Less is known whether the quality of embryos might already be impaired at onset of cancer.

Unfortunately, we were not able to study intention to treat in this context since patients who started the fertility preservation procedure without success (no oocytes or embryos available for cryopreservation) were not included in this study. Furthermore, we were not able to extract any demographic information from our data, other than age and parity. Given the fact that 10 out of 12 centers in the Netherlands participated in the study, we conclude that our data is generalizable to the general public, especially in countries with similar insurance regulations. Of note though, even in settings with equal access, highly educated women are overrepresented (Marklund *et al.*, 2021).

Slowly more data on female fertility preservation procedures are being reported (Rodriguez-Wallberg *et al.*, 2019; Ter Welle-Butalid *et al.*, 2019; Vriens *et al.*, 2020; Cobo *et al.*, 2021; Marklund *et al.*, 2021; Tsafrir *et al.*, 2022; Loreti *et al.*, 2024). Our study is one of the larger studies conducted in the field of female fertility preservation and contributes to the information on the utilization and success rates of cryopreserved oocytes and embryos in the context of fertility preservation for different indications. This information is important for the professional to share with the patient during counseling so they can use it for their final decision making. One factor cited as reducing the risk of decision regret is having more accurate information about success rates (Greenwood *et al.*, 2018). Given the size of our data, this can contribute to more accurate counseling on success rates and therefore reduce decisional regret. With the growing insights on this topic, including our data, an evidence-based decision tool could be developed that summarizes the risk of infertility, the chances of natural conception (or success of fertility treatments) after fertility preservation versus the utilization rate and success rates of fertility preservation. Such a tool could help with decision making pros or cons of fertility preservation, as well as with decision making on mode of conception once there is an active desire to conceive.

Furthermore, the data from this study can be used to update the guidelines on female fertility preservation. Although the difference was not significant, our results add to the evidence that success rates are lower for those with oncological indications.

In conclusion, after 10 years of follow-up, a quarter of women who had undergone fertility preservation had returned to use their frozen oocytes or embryos. A median of two embryo transfers was performed per patient in a median follow-up duration of 42 months, which resulted in a cumulative live birth rate of 35% per patient. The key figures from this population-based study may support the counseling of future patients.

## Supplementary Material

deae243_Supplementary_Figure_S1

## Data Availability

The data underlying this article will be shared upon reasonable request to the corresponding author with permission of all participating centers.
