# Supplementary material for: Outcomes of female fertility preservation with cryopreservation of oocytes or embryos in the Netherlands: a population-based study
Source: Hum Reprod. 2024 Oct 30;39(12):2693–701. doi: 10.1093/humrep/deae243 (PMC11630040; doi:10.1093/humrep/deae243)
Supplement: deae243_Supplementary_Figure_S1 [file deae243_supplementary_figure_s1.pdf]

OXFORD
human reproduction
Human Reproduction, 2024, 1–1
https://doi.org/10.1093/humrep/deae243
Supplementary Data

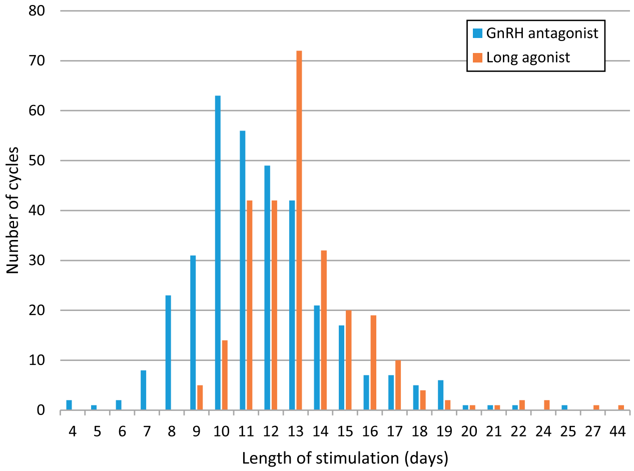

Supplementary Figure S1. Distribution of length of stimulation in days of GnRH antagonist protocols and long agonist protocols since 2016. Before 2016, the stimulation protocol was not often reported, therefore only stimulations since 2016 are included.

© The Author(s) 2024. Published by Oxford University Press on behalf of European Society of Human Reproduction and Embryology.
This is an Open Access article distributed under the terms of the Creative Commons Attribution License (https://creativecommons.org/licenses/by/4.0/
), which permits unrestricted reuse, distribution, and reproduction in any medium, provided the original work is properly cited.
Human Reproduction, 2024, 00, 1–10
https://doi.org/10.1093/humrep/deae243
Original Article
